# Inhibition of phosphodiesterase: A novel therapeutic target for the treatment of mild cognitive impairment and Alzheimer’s disease

**DOI:** 10.3389/fnagi.2022.1019187

**Published:** 2022-10-04

**Authors:** Jianwen Sheng, Shanjin Zhang, Lule Wu, Gajendra Kumar, Yuanhang Liao, Pratap GK, Huizhen Fan

**Affiliations:** ^1^Department of Gastroenterology, The People’s Hospital of Yichun City, Yichun, China; ^2^Department of Neuroscience, City University of Hong Kong, Kowloon Tong, Hong Kong SAR, China; ^3^Department of Biochemistry, Davangere University, Davangere, India

**Keywords:** natural products, phosphodiesterase, Alzheimer’s disease, cyclic-AMP, Vinpocetine

## Abstract

Alzheimer’s disease (AD) is the most common form of dementia and is ranked as the 6th leading cause of death in the US. The prevalence of AD and dementia is steadily increasing and expected cases in USA is 14.8 million by 2050. Neuroinflammation and gradual neurodegeneration occurs in Alzheimer’s disease. However, existing medications has limitation to completely abolish, delay, or prevent disease progression. Phosphodiesterases (PDEs) are large family of enzymes to hydrolyze the 3’-phosphodiester links in cyclic adenosine monophosphate (cAMP) and cyclic guanosine monophosphate (cGMP) in signal-transduction pathways for generation of 5’-cyclic nucleotides. It plays vital role to orchestrate several pharmacological activities for proper cell functioning and regulating the levels of cAMP and cGMP. Several evidence has suggested that abnormal cAMP signaling is linked to cognitive problems in neurodegenerative disorders like AD. Therefore, the PDE family has become a widely accepted and multipotential therapeutic target for neurodegenerative diseases. Notably, modulation of cAMP/cGMP by phytonutrients has a huge potential for the management of AD. Natural compounds have been known to inhibit phosphodiesterase by targeting key enzymes of cGMP synthesis pathway, however, the mechanism of action and their therapeutic efficacy has not been explored extensively. Currently, few PDE inhibitors such as Vinpocetine and Nicergoline have been used for treatment of central nervous system (CNS) disorders. Considering the role of flavonoids to inhibit PDE, this review discussed the therapeutic potential of natural compounds with PDE inhibitory activity for the treatment of AD and related dementia.

## Graphical Abstract

PDE inhibitors for the treatment of Alzheimer’s disease. 
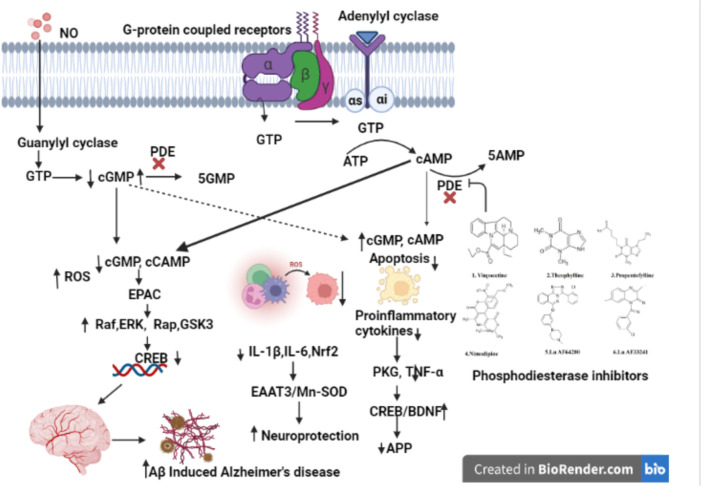

## Introduction

Alzheimer’s disease (AD) is a neurodegenerative cognitive disorder affecting around 36 million individuals around the world. By 2050, this number is expected to quadruple (Ribaudo et al., 2020). AD is characterized by memory loss, inability to learn, prohibiting their normal communication, prevent daily activity over the time and significantly reduces the patients’ quality of life (Pratap et al., 2017). Cholinergic transmission and glycation dysfunction, amyloid formation, and oxidative stress have been implicated for disease’s onset and progression (Ribaudo et al., 2021). Cholinesterase inhibitors have been approved by Food and Drug Administration (FDA, USA) for the treatment of mild to moderate AD. Memantine, an N-methyl-D-aspartate receptor non-competitive antagonist, is another treatment option for moderate to severe AD (Geerts and Grossberg, 2006). “Disease-modifying” drugs prevents or at least effectively modify the course of AD, are still under investigations (Munoz-Torrero, 2008). These drugs interfere with the pathogenesis factors responsible for clinical symptoms such as production of extracellular amyloid plaques, intracellular neurofibrillary tangles, inflammation, oxidative damage, cholesterol metabolism and prevent the disease progression (Parihar and Hemnani, 2004). Currently available drugs do not cure AD; however, symptoms are alleviated for a brief time. Therefore, it is imperative to explore the novel targets and therapeutics agents from natural compounds for treatment of AD and dementia (Yiannopoulou and Papageorgiou, 2013).

Phosphodiesterases (PDEs) are intracellular enzymes and recently gained attention as potential therapeutic targets for the treatment of cognitive loss in aging and AD (Heckman et al., 2017). The PDE family has been reported as multipotential target in several neurological disorders (Nabavi et al., 2019). PDEs degrades cyclic nucleotides, e.g., cyclic-AMP (cAMP) and cyclic-GMP (cGMP). The role of PDEs in cognitive enhancement has recently been discovered in a transgenic fly model of learning defects (Tibbo et al., 2019). Abnormal cAMP and/or cGMP signaling are associated with cognitive impairment in neurodegenerative disorders (Di Benedetto et al., 2021). Dysfunction of signal transduction in disease condition are caused by abnormal PDE function, resulting uncoordinated cAMP and cGMP in memory related brain areas, enhanced Aβ production and disrupt the memory formation (Nabavi et al., 2019). Memory loss tends to occur before nerve cell death in AD, suggesting that neuronal dysfunction as key pathological factor. Therefore, PDE inhibitors could be postulated to improve AD symptoms by restoring synaptic function due to restoration of cyclic AMP response element-binding protein (CREB) signaling pathway (García-Osta et al., 2012).

PDEs have been emerging as interesting targets for the treatment of neurodegenerative disease such as AD. Some small molecule compounds targeting PDE and its isoforms could be an effective anti-Alzheimer’s agents (Heckman et al., 2017; Ribaudo et al., 2021). In this review, we discussed the mechanisms of PDE inhibitors (PDE-Is) of natural and synthetic compounds based on the currently available literature with preclinical and clinical data.

## Methodology

Online literature was retrieved by using well-known scientific search engines such as Google Scholar, PubMed, Elsevier journal, EMBASE, Science Direct, Book chapters, Springer Link, Elsevier, Taylor and Francis, ACS, Wiley publishers and scientific literature, as well as reports and documentation from government organizations, were assessed. The results were cross-referenced, resulting in a total of 144 references listed in this review, spanning the years 1996–2022. The current review discussed the therapeutic potential of PDE inhibitor in mild cognitive impairment (MCI) and AD. Tables 1–3 shows the natural and synthetic compounds and mode of action in the inhibition of PDE.

**TABLE 1:** Human PDE families.

PDE families	Gene	Feature
PDE1	PDE1A	Calcium/calmodulin regulated
	PDE1B	
	PDE1C	
PDE2		cGMP and cAMP specific
PDE3	PDE3A	cGMP inhibited
	PDE3B	
PDE4	PDE4A	cAMP specific
	PDE4B	
	PDE4C	
	PDE4D	
PDE5	PDE5A	cGMP specific
PDE6	PDE6A	Photoreceptor
	PDE6B	
	PDE6C	
PDE7	PDE7A	Rolipram insensitive
	PDE7B	
PDE8	PDE8A	cAMP specific
	PDE8B	
PDE9	PDE9A	cGMP specific
PDE10	PDE10A	cAMP inhibited, dual substrate
PDE11	PDE11A	Dual substrate

**TABLE 2:** List of FDA approved drugs as PDE inhibitors with a focus on Alzheimer’s disease.

Drug	Targeted enzyme	Application	Side effects
Vinpocetine	PDE1	Improve memory in people with mild cognitive impairment (MCI) (Prickaerts et al., 2017).	Flush, nausea, dizziness, dry mouth, transitory hypo- and hypertension, headaches, heartburn, and low blood pressure are among side effects of Vinpocetine (Prickaerts et al., 2017; Dubey et al., 2020).
Cilostazol	PDE3	Reduce the cognitive decline in AD (Prickaerts et al., 2017).	Headache, palpitations and diarrhea (Weintraub, 2006)
Sildenafil, vardenafil and tadalafil	PDE5 and 6	In several AD mice models, it enhances cognition and lowers hippocampus Aβ burden (Prickaerts et al., 2017).	Headache, face flushing, nasal congestion, and dyspepsia are some of the negative effects (Prickaerts et al., 2017).
Tadalafil	PDE11		

**TABLE 3:** List of natural products for PDE inhibitors and their impact on cognitive enhancement in clinical research, with a focus on Alzheimer’s disease.

Phytochemical compound	PDE type	Type of compound	Effect
6-gingerol	PDE4D	Polyphenols	Cox-2 expression is inhibited by blocking p38 mitogen-activated protein (map) kinase and nf-b activation (Azam et al., 2014; Furlan and Bren, 2021).
Amentoflavone	PDE3	Biflavonoid	Inhibition of phosphodiesterase (PDE) reduces camp destruction (Dell’Agli et al., 2006; Xiong et al., 2021)
Apigenin	PDE4	Flavonoids	Inhibit the phosphodiesterase enzyme (PDE) (Wang et al., 2018a).
Beta carboline	PDE1	Alkaloid	Beta act as dual inhibitors of AChE and PDEs (Ribaudo et al., 2021).
Caffeine	PDE5	Flavonoids	Caffeine was discovered to be a non-selective PDE inhibitor, inhibiting both cgmp-specific and PDE type 5 PDEs (PDE5) (Prickaerts et al., 2017).
Capsaicin	PDE4D	Polyphenols	Anti-AD (Furlan and Bren, 2021).
Curcumin	PDE4D	Polyphenols	PDEs (enzymes that convert cyclic AMP and cyclic GMP into 5’ AMP and 5’-GMP) were downregulated in response to curcumin therapy (Heckman et al., 2015; Kim and Clifton, 2018; Furlan and Bren, 2021).
Epigallocatechin-3- gallate	PDE4	Polyphenols	EGCG reduced sevoflurane-induced downregulation of camp/CREB and BDNF/trkb signaling (Ding et al., 2017).
Ferulic acid	PDE4B2	Phenolic compound	FA boosted intracellular camp levels while decreasing intracellular Ca^2+^ levels. FA can decrease PDE4B2 activity, according to the docking data (Huang et al., 2016).
Ginsenoside rg1	PDE	Class of steroid glycosides, and triterpene saponins	The activity of camp-dependent phosphodiesterase (camp-PDE) was dramatically reduced by Rg1, which increased intracellular camp levels (Stancheva and Alova, 1993; Francis et al., 2011; Min Lai et al., 2018; Mohamed et al., 2019).
Glycocoumarin	PDE3 and4	Coumarin	Glycocoumarin is a non-specific phosphodiesterase inhibitor (PDEs) (Sato et al., 2006).
Icariin	PDE5	Flavonoid	Icariin is a putative selective dual-target ache/PDE5 inhibitor that could be used to treat Alzheimer’s disease. Possess significant anti-AD properties in an indifferent of AD mouse models (Mao et al., 2018).
Luteolin	PDE-1, 4 and 5	Flavonoids	Inhibit the phosphodiesterase enzyme (PDE) (Ayoub and Melzig, 2006; Yu et al., 2010).
Physostigmine	PDE	Alkaloid	Substantial camp PDE inhibition (Curley et al., 1984; Pratap et al., 2021).
Quinovic acid	PDE1	Glycosides	Quinovic acid and its few derivatives have an inhibitory effect against the enzyme phosphodiesterase-1 (Mostafa et al., 2006).
Resveratrol	PDE4D	Polyphenols	Resveratrol may involve the regulation of neuronal inflammation and apoptosis *via* PDE4 subtypes related camp-CREB-BDNF signaling (Wang et al., 2016; Furlan and Bren, 2021).
Sophoflavescenol	PDE4, 5, and 9	Flavonol	Inhibitors that target cGMP (Ribaudo et al., 2021).
Withanolides	PDE4D	Steroids	PDE4D was discovered to be the most potent target for withanolides after molecular docking, molecular dynamics modeling, and free energy calculations (Rathi and Sundar, 2022).

## Phosphodiesterases and their inhibitors

PDEs (cyclic nucleotide phosphodiesterases) are enzymes to catalyze the hydrolysis of cAMP and cGMP and terminate cyclic nucleotide signaling (Zuo et al., 2019). PDEs are classified into 11 subtypes based on their structural and functional characteristics (PDE1-PDE11) (Amin et al., 2018). Inhibition of PDEs, activation of AC/cAMP/PKA, or NO/cGMP/PKG (Francis et al., 2010) signaling pathways increases cAMP and cGMP levels in the brain by increasing the level of the CREB, improving the synaptic transmission, and lower cognitive deficits (Bollen et al., 2014). Preclinical models and clinical studies of AD have reported that PDE inhibitors such as PDE1, PDE2, PDE4, PDE5, PDE9, and PDE10 improve the cognitive deficits (Zhang et al., 2018). Several PDE subfamilies are abundantly expressed in the human brain, suggesting the association of PDE inhibition in neurodegenerative processes through regulation of cAMP and/or cGMP levels. PDEs are currently considered to be attractive targets for the treatment of Alzheimer’s disease, as several PDE inhibitors have been proven to improve cognitive function (Wu et al., 2018). PDE antagonists has also been suggested as multi-target medications for neurodegenerative disorders (González et al., 2019). It has potential to cross the blood–brain barrier and attain optimum inhibitory concentrations in the central nervous system (CNS) to exert therapeutic effects. Several molecules and available medications, such as Sildenafil, Vardenafil, and Tadalafil have been exploited as PDE inhibitors in AD treatment (Ribaudo et al., 2020).

PDEs are categorized into three class: PDEI, PDEII, and PDEIII. Class I PDEs in mammals have half HD domain in the C-terminal and shows a strong affinity for cAMP and/or cGMP (Omori and Kotera, 2007). Half of N-terminal region of the protein regulates PDE enzymatic activity and subcellular localization. Protein kinases targeting phosphorylation sites and lipid modification sites are present in some PDEs (Omori and Kotera, 2007; Lorigo et al., 2022).

In humans, rats, and mice, twenty-one different class I PDE genes have been discovered. They are divided into 11 groups (Table 1) based on structural similarities like sequence homology, protein domains, and enzymatic features including sensitivity to endogenous regulators and inhibitors (Puzzo et al., 2008). The C-terminal catalytic region contains around 270 amino acids are conserved among PDE families, with 35–50% identical sequence (Fisher et al., 1998). Some PDE families are made up of two to four subfamily genes that share more than 70% sequence and have the same protein domain organization (Degerman et al., 1997; Epstein, 2017; Gu et al., 2022).

### Characteristics of phosphodiesterases gene families

PDEs are important regulators of intracellular cAMP and cGMP concentrations to modulate their signaling pathways and physiologic effects (Lugnier, 2006; Peiró et al., 2011). There are more than 20 genes in mammals to encode over 50 distinct PDE proteins that are presumably produced in mammalian cells, with each family including one to four genes (Bender and Beavo, 2006; Lugnier, 2006). PDE1 to PDE6 isoforms were first well-characterized due to their prevalence in many tissues and cells, their unique contribution to tissue function and regulation in pathophysiology such as inflammation, neurodegeneration, and cancer (Lugnier, 2006). However, the role of newly identified PDE7 to PDE11 families have not been determined (Pdes et al., 2014). The potential roles of PDE1-11 in signaling pathways and their targets have been discussed below.

Phosphodiesterase 1: It resembles PDEs encoded by PDE1A to -C and known to hydrolyze cAMP and cGMP in the presence of Ca^2+^ /CaM. PDE1A has a strong affinity for cGMP in humans (Loughney et al., 1996; Omori and Kotera, 2007).

Phosphodiesterase 2: phosphodiesterase 2 (PDE2A) hydrolyzes both cGMP and cAMP at same rates (Pavlaki and Nikolaev, 2018). cGMP binding to PDE2A’s GAF domain allosterically stimulates for mutual control of cAMP and cGMP signaling (Omori and Kotera, 2007).

Phosphodiesterase 3: PDE3A and PDE3B are subfamily genes of PDE3, a protein that binds to both cAMP and cGMP with a high affinity (Heikaus et al., 2009). PDE3s are known as cGMP-inhibited cAMP PDEs (Degerman et al., 1997). The PDE3 family is distinguished by the presence of a 44-amino acids inserted in the catalytic domain. The presence of N-terminal hydrophobic membrane attachment regions (NHRs) is another distinguishing feature (Omori and Kotera, 2007).

Phosphodiesterase 4: PDE4A to-D are four closely related subfamily genes that encode cAMP-specific Rolipram-sensitive PDEs (Table 1). Splice variants in the PDE4 family are divided into three N-terminal variant groups depending on the presence or absence of N-terminal Upstream Conserved Regions (UCR) domains (Omori and Kotera, 2007).

Phosphodiesterase 5: PDE5A has two GAF domains in its half N-terminal and hydrolyzes cGMP selectively. This enzyme’s allosteric binding to cGMP domain suggests PDE5A as a cGMP-binding cGMP-specific PDE (Heikaus et al., 2009). The activation of PDE5A enzyme is linked to PKG and PKA-dependent phosphorylation site in the N-terminal region. cGMP binding to PDE5A/GAFA phosphorylation is promoted by a domain, which enhances the catalytic function along with increased the cGMP binding affinities (Omori and Kotera, 2007; Ahmed et al., 2021; Gu et al., 2022).

Phosphodiesterase 6: The level of cGMP, a second messenger in visual signal transduction, is closely controlled in retinal rod and cone cells by three PDE6 subfamily genes that govern cGMP hydrolysis. PDE6 activity is regulated by inhibitory subunits, which is a unique feature of this PDE family (Cote, 2006). Sildenafil, and vardenafil, but not tadalafil, have lower affinity for inhibiting PDE6 than PDE5A (Omori and Kotera, 2007; Wilkins et al., 2008).

Phosphodiesterase 7: PDE7A and PDE7B are Rolipram insensitive, high-affinity cAMP-specific PDEs. PKA pseudo substrate site exists at the N terminus of the PDE7A subfamily. Dipyridamole has a non-selective inhibitory effect on PDE7 activity (Ahlström, 2001; Omori and Kotera, 2007).

Phosphodiesterase 8: PDE8 is divided into two subfamilies: PDE8A and PDE8B. PDE8s are cAMP-specific PDEs with N-terminal REC (cheY-homologous receiver) and PAS (per-arnt-sim) domains that are resistant to Rolipram and 3-isobutyl-1-methylxanthine (IBMX) (Omori and Kotera, 2007).

Phosphodiesterase 9: PDE9A has a high affinity for cGMP hydrolysis. However, there are no evidence of PDE9A activity regulation or the presence of endogenous PDE9A activity in tissue or cell extracts (Omori and Kotera, 2007).

Phosphodiesterase 10: PDE10A hydrolyzes both cAMP and cGMP and has two GAF domains at the N-terminus (Soderling et al., 1999). cAMP stimulates the enzymatic activity of a chimeric PDE10A GAF domain and cyanobacterial adenylyl cyclase, suggesting that cAMP may acts as an allosteric modulator of PDE10A activity (Omori and Kotera, 2007; Schultz and Natarajan, 2013).

Phosphodiesterase 11: PDE11A4 is a full-length version with two GAF domains and a catalytic domain. In tissue or cell extracts, PDE11A activity has yet to be confirmed. Tadalafil has been proven to suppress PDE11A activity and with less potency than PDE5A (Omori and Kotera, 2007; Schultz and Natarajan, 2013).

### Role of phosphodiesterases and their inhibitors in Alzheimer’s disease

**FIGURE 1:**
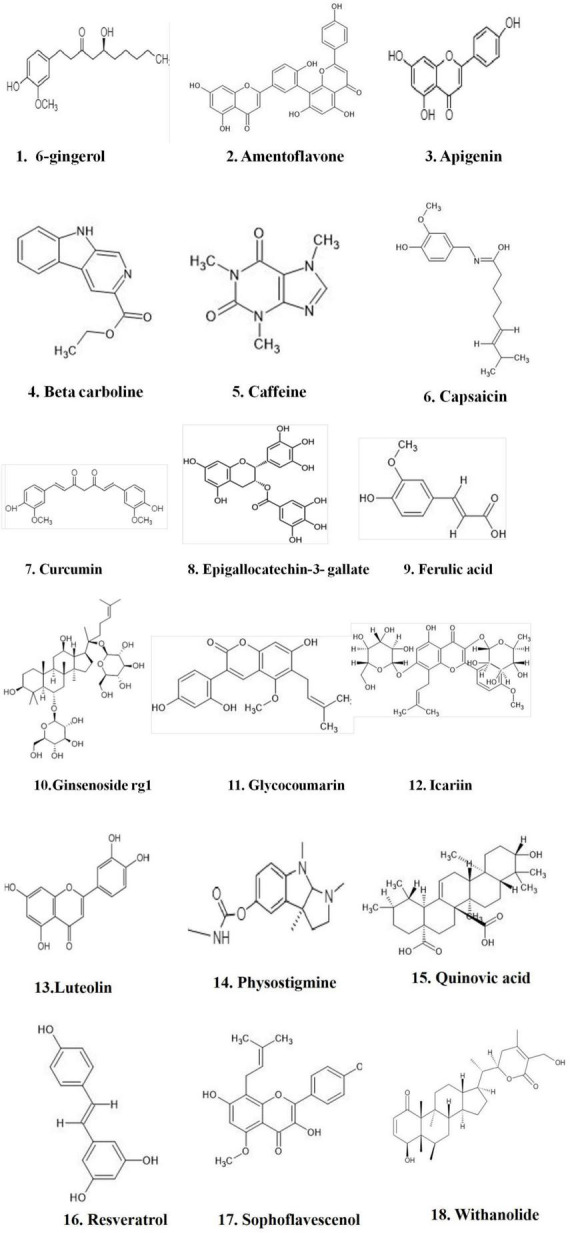
Natural compounds used for PDE inhibitors for the treatment of AD. The figure is original and generated by Bio-render software.

Phosphodiesterases (PDEs) are important regulators of cyclic nucleotide-mediated signaling levels (Barnes, 1995). cAMP and cGMP signaling have been linked to neuroplasticity and protection (Figure 1). Usage of PDEs inhibitors to manipulate cAMP and cGMP levels in the cell has become a popular strategy for treatment of various neurodegenerative disorders such as AD (Bollen and Prickaerts, 2012; Hesse et al., 2017).

Cognitive impairment has been associated with synthesis, execution, and/or degradation of cyclic nucleotides. In terms of cognition, cyclic nucleotides (cAMP and cGMP) play an important role in gene transcription, neurogenesis, neural circuitry, synaptic plasticity, and neuronal survival (Cardinale and Fusco, 2018) and animal studies have shown the plasticity of synapses and cognition deficit. Animal studies also reports that increasing cGMP-PKG signaling increases long-term memory and required cAMP-PKA (Bollen et al., 2014) signaling. Impairments in these basic processes are underlying cause of cognitive impairment observed in AD patients, implying that cAMP/cGMP signaling plays a critical role in AD patients (Sharma et al., 2020). For example, the AC/cAMP/PKA signaling pathway is downregulated in AD patients. Phosphorylation of tau by cAMP and its main target protein kinase, PKA, is hypothesized to play a role in the genesis of neurofibrillary tangles (Sanders and Rajagopal, 2020). Hence, downregulation of AC/cAMP/PKA signaling pathway, which is also a major activator of CREB, can explain loss of synaptic plasticity and memory impairment in AD (Bollen and Prickaerts, 2012; Argyrousi et al., 2020). Some studies have reported the changes in the expression of cAMP-specific PDE mRNAs in AD brains (Pérez-Torres et al., 2003). In the early stages of AD, increased expression of PDE4A, PDE4B, and PDE7A has also been found (Bollen and Prickaerts, 2012).

### Mechanism of action of phosphodiesterase inhibitors

A cyclic nucleotide is a type of nucleotide that has phosphodiesterases (PDEs), which are crucial enzymes in the intracellular signal transduction cascade that occurs when membrane-bound receptors are activated (Ahmad et al., 2015; Preedy, 2020). Second messengers such as cyclic nucleotide (cAMP/cGMP) have been shown to have an important part in the control of cellular functions like signal transduction and synaptic transmission of numerous neurotransmitters in the brain (Gorshkov and Zhang, 2014). Protein kinase A (PKA) and Protein kinase G (PKG) are two target enzymes for cyclic nucleotides such as cAMP/cGMP (Kumar et al., 2015). Cyclic nucleotide mediated transactivation of CREB and brain-derived neurotrophic factor (BDNF) has been reported to have a key impact on cognitive processes (Nair and Vaidya, 2006). CREB is an activity-inducible transcription factor that is activated by numerous kinases binding to the Serine-133 (Ser-133) region. Synaptic plasticity, neuronal growth, and development are enhanced by CREB-mediated transcriptional activity (Nair and Vaidya, 2006). In the conventional model, adenylyl cyclase (AC) or guanylyl cyclase (GC) generate cAMP or cGMP at the plasma membrane in response to external signal, diffuse throughout the cell, where they interact with specific effector proteins to govern a variety of cellular activities (Bolger, 2021).

**FIGURE 2:**
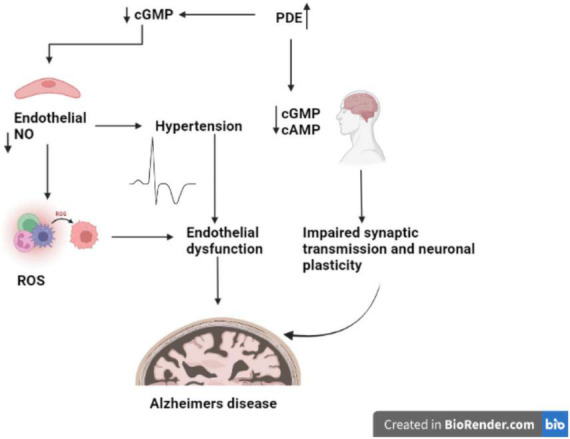
Role of PDE in AD. The figure is original and generated by Bio-render software.

The changes in the expression of PDE1, PDE4, PDE9, and PDE10 in brain tissues are linked to AD (Kumar et al., 2015; Figure 2). PDE4, a cAMP-specific PDE (García-Osta et al., 2012; Li et al., 2018), PDE9, a cGMP-specific PDE, and the dual cAMP and cGMP specific enzymes PDE1 and PDE10 (Reyes-Irisarri et al., 2007) have been reported to significantly expressed in the AD brain. The PDEs regulate spatial and temporal features of cyclic nucleotide signaling by inactivating cAMP and cGMP through metabolic in activation (Liu et al., 2008). Thus, targeting PDEs to increase synaptic function, or synaptic resilience, could be an effective strategy to treat AD. Furthermore, data from several pre-clinical investigations in AD experimental models have shown that inhibiting PDEs is beneficial (Kumar et al., 2015; Saxena, 2016; Mondal et al., 2017).

PDEs play a crucial role to exerts pharmacological activities, including cell function, by regulating the levels of cAMP and cGMP. The PDE family has been a popular multipotential target to explore a variety of disease pathologies (Nabavi et al., 2019). It has been reported that abnormal cAMP signaling is linked to cognitive impairment in neurodegenerative disorders like Alzheimer’s (Di Benedetto et al., 2021). Disease-related signal transduction dysregulation are caused by abnormal PDE function caused by uncoordinated cAMP responses enhances the Aβ production in brain regions and disrupts the memory formation and (Figure 2; Tibbo et al., 2019). Memory loss tends to occur before nerve cell death in AD, suggesting that neuronal dysfunction as root cause of disease’s pathology (Tian et al., 2014).

Phosphodiesterase inhibitors blocks the phosphodiesterase enzymes PDE-3, PDE-4, and PDE-5 and prevent the degradation of cGMP or cAMP, increases their levels in smooth muscle cells, and induces relaxation and vasodilation in target cells (Padda and Tripp, 2021). Chronic obstructive pulmonary disease (COPD), benign prostatic hyperplasia (BPH), acute decompensated heart failure, psoriasis, psoriatic arthritis (PA), Alzheimer’s disease, atopic dermatitis, and newborn apnea are treated by phosphodiesterase inhibitors (Mannhold et al., 2014). Smooth muscle relaxation, vasodilation, and bronchodilation by PDE inhibitor prevents cAMP and/or cGMP breakdown (Padda and Tripp, 2021).

**FIGURE 3:**
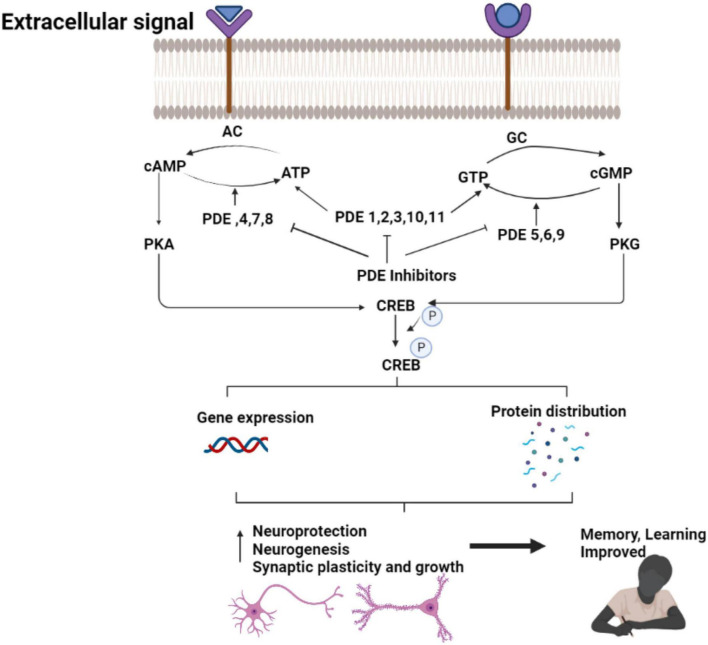
Therapeutic effect of PDE inhibitors in AD. The figure is original and generated by Bio-render software.

Specific phosphodiesterase (PDE) inhibitors have been shown to improve memory function in a variety of animal models of AD. PDE inhibitors stimulate gene transcription by activating the CREB (Fiorito et al., 2018; Figure 3). Long-term memory formation and persistent long-term potentiation (LTP) measuring the synaptic plasticity and strength, are driven by CREB-dependent gene expression (Davis et al., 2000). It occurs in the hippocampus through the development of new synaptic connections. Memory loss appears to occur before nerve cell death in AD, suggesting that neuronal dysfunction could be responsible for the pathophysiology of early-stage AD (Abraham, 2019).

The CREB pathway is one of the signaling pathways associated with neuronal plasticity. The brain’s CREB pathway reacts to increased calcium due to neuronal activity. Protein kinase A (PKA), protein kinase C (PKC), the calcium/calmodulin-dependent protein kinases CaMKII and CaMKIV, the extracellular signal-regulated kinase (ERK)-activated kinases mitogen- and stress-activated protein kinase phosphorylates CREB directly (MSK) (García-Osta et al., 2012; Wang et al., 2018a).

The enhancements in memory and learning in FAD mice has been shown after increased CREB signaling, while impaired CREB signaling cause AD symptoms and cognitive deficits. Phosphodiesterases (PDE), a class of enzymes that hydrolyze cAMP (PDE4, PDE7, PDE8), cGMP (PDE5, PDE6, PDE9), or both cAMP and cGMP (PDE1, PDE2, PDE3, PDE10, PDE11) maintain the normal CREB signaling. The strategy to pharmacologically boost CREB signaling could be a potential treatment for cognitive dysfunction in AD by decreasing the expression or activity of PDEs (Bartolotti and Lazarov, 2019).

PDE inhibitors alleviates AD symptoms by restoring synaptic function *via* stimulation of the CREB signaling pathway (Wang et al., 2018b). Furthermore, other CREB-independent pathways appear to work in tandem to repair cognitive impairment in AD.

### Cyclic nucleotide signaling and neuroinflammation

Increased activation of microglia and astrocytes, activated complement proteins, cytokines, and reactive oxygen, nitrogen, and carbonyl species have been shown to linked with AD inflammatory response. Chronic neuroinflammation has been recognized as one of the primary causes of AD (Millington et al., 2014). TNF and IL-1 like inflammatory cytokines increases the production of adhesion molecules on endothelial cells, which bind to leukocyte ligands and allow activated leukocytes to enter the CNS (Strang et al., 2020). Endothelial cells excrete chemokines in response to inflammation, which attract leukocytes to the CNS. Immune cell trafficking across the BBB may start or contribute to a “vicious circle” in the pathological process, resulting in progressive synaptic and neuronal dysfunction and neuronal death in diseases like AD (Peixoto et al., 2015). cGMP is a key mediator of the activity of nitric oxide (NO) and natriuretic peptides in the CNS (Schlossmann and Hofmann, 2005). NO is a gaseous free radical produced by intracellular isoforms of the nitric oxide synthase enzyme and serves as a crucial marker of intra- and extracellular activities. The expression of inflammatory mediators is orchestrated by inflammatory response and regulates the physiological process (Akiyama et al., 2000). Inhibition of IκB degradation due to restriction of IKK activity by cAMP/PKA or increased quantities of resynthesized IB, cAMP interferes with the function of the proinflammatory transcription factor, Nuclear Factor-kappa B (NF-κB). NF-κB activates gene expression for a variety of inflammatory and immune mediators (Peixoto et al., 2017). Stimuli like proinflammatory cytokines, B- and T-cell activators, pathogen-associated molecular patterns (PAMPs), and oxidative stress, cyclic AMP modulates NF-κB. It also affected by less common stimuli like amyloidogenic peptide, thrombin, and high glucose levels (Akiyama et al., 2000; Peixoto et al., 2015; Sanders and Rajagopal, 2020). The NO/cGMP/PKG system play critical role to inhibit the activation of a proapoptotic pathway, allowing brain cells to survive. This neuroprotective process is relevant during ischemia, inflammation, or trauma to the brain. The CREB, a transcription factor involved with regulation of neurotransmitters, growth factors, and other signaling molecules are essential for long-lasting changes in synaptic plasticity, mediating the conversion of short-term memory to long-term memory and neuronal survival in retinal neuroglial progenitor cells, activates the NO/cGMP/PKG antiapoptotic cascade in retinal neuroglial progenitor cells (Akiyama et al., 2000; Peixoto et al., 2015, 2017; Yougbare, 2021).

### Current status of phosphodiesterases inhibitors for the treatment of Alzheimer’s disease

PDEs expression in the brain are the focus of current research due to its critical role on regulation of neuroinflammation as discussed above. Based on the genetic variations in patients with neurodevelopmental disorders, animal model phenotypes (Gawel et al., 2020), current research is focused on the pharmacological effects of PDE inhibitors, a medication class that is rapidly evolving and becoming more widely used for the treatment of brain disorders. Pfizer laboratories synthesized UK-92,480 (Sildenafil citrate) compound in 1989, indicating its efficacy as a PDE5 inhibitor. However, multiple studies on healthy volunteers were investigates to study the pharmacokinetics, pharmacodynamics, and tolerance of UK-92,480 revealed side effects including flushing, muscle aches, indigestion, and headaches (Ghofrani et al., 2006).

After a few modifications, sildenafil was approved as the first PDE5 inhibitor for the treatment of erectile dysfunction (ED) in 1998. Vardenafil was approved in 2003, giving patients another option (Huang and Lie, 2013). In comparison to other PDE5 inhibitors, Avanafil was approved by the US Food and Drug Administration in 2012 and the European Medicines Agency in 2013. It was marketed under the brand name Stendra or Spedra and had a short half-life and a rapid onset of action (Bourin, 2018). The NO/cGMP pathway is involved in numerous physiological activities such as the urogenital, pulmonary, and gastrointestinal systems, as well as the central nervous system, Alzheimer’s disease, and the pathophysiology of a wide range of disorders. Despite of typical side effects shown by current drugs like sildenafil, Vardenafil, and Tadalafil (Tables 2, 4), these are most effective oral therapies for treating erectile dysfunction and AD (Ayoub and Melzig, 2006; Prickaerts et al., 2017). However, serious side effects such as non-arteritic hearing loss, dyspepsia, migraines, and priapism have also been recorded (Yafi et al., 2018). As a result, alternate sources of PDE inhibitors are required to eliminate or decrease the side effects of synthetic PDE inhibitors (Anand Ganapathy et al., 2021). Natural or traditional herbs are considered as most desirable alternative sources for the development of new active pharmacophores since they have demonstrated bioactivities with potential utility for health improvement as well as less side effects than synthetic medications (Pratap et al., 2017; Pratap and Shantaram, 2020).

**TABLE 4:** List of synthetic drugs for PDE inhibitors and their impact on clinical significance with a focus on Alzheimer’s disease.

FDA approved drug	PDE type	Clinical significance
Vinpocetine	PDE1	Improve memory in people with mild cognitive impairment (MCI), dementia, memory loss (Prickaerts et al., 2017).
Theophylline	PDE	Theophylline is a non-selective phosphodiesterase (PDE) inhibitor (Chen et al., 2008).
Propentofylline	PDE	Propentofylline is used to treat canine cognitive impairment, which is caused by age-related wild-type Aβ deposition, similar to Alzheimer’s disease. Phosphodiesterase inhibitors may help to prevent and treat Alzheimer’s disease (Sanders and Rajagopal, 2020).
Nimodipine	PDE1 and 2 inhibitors	Nimodipine is a dihydropyridine that inhibits PDE1 and antagonizes/blocks primarily L-type Ca^2+^ channels (Lugnier, 2006).
Lu AF64280	PDE2 and 10 inhibitors	Lu AF64280 is a new phosphodiesterase (PDE) 2A inhibitor that is brain penetrant and selective. *In vivo* models/tests relevant to cognitive processing or antipsychotic-like effects, as well as *in vitro/in vivo* assays indicative of PDE2A inhibition (Redrobe et al., 2014).
Lu AF33241		Lu AF33241, a new brain-penetrant phosphodiesterase inhibitor of (PDE) 2A and 10A tool compound, *in vivo* models/tests related to cognitive processing and antipsychotic-like activity, and *in vitro*/*in vivo* assays indicative of PDE2A and/or PDE10A inhibition (Redrobe et al., 2014; John et al., 2015).
Cilostazol	PDE 3 inhibitor	In a mouse model of Alzheimer’s disease, cilostazol, a selective inhibitor of phosphodiesterase (PDE) 3, promotes amyloid β clearance and alleviates cognitive deficits (Tsuji et al., 2020).
Rolipram	PDE4 inhibitor	Rolipram, a phosphodiesterase-4 inhibitor, was studied in mice to see if it could help with cognitive deficiencies caused by streptozotocin and normal aging. It may improve with memory problems due to its anti-cholinesterase, anti-amyloid, anti-oxidant, and anti-inflammatory properties (Kumar and Singh, 2017).
Zaprinast	PDE5 inhibitor	Prickaerts et al. (1997) found that the PDE5 inhibitor zaprinast significantly increased performance in an ORT while not affecting peripheral vascular function (Prickaerts et al., 1997; Rutten et al., 2009).
Sildenafil/Tadalafil	PDE 5	The PDE5 inhibitor sildenafil has powerful anti-AD benefits, reversing cognitive decline (García-Osta et al., 2012). The capacity of PDE5 inhibitors to increase cGMP levels and so interfere with the NO/cGMP/PKG/CREB signaling pathway has led to the concept that PDE5 inhibition could be employed as a viable therapeutic method for the treatment of AD (Zuccarello et al., 2020).
Zaprinast, Dipyridamole Vardenafil	PDE 6	Transducin-activated (Ghosh et al., 2009).
Dipyridamole, Thiadiazole	PDE 7	Rolipram-insensitive, IBMX-insensitive (Ghosh et al., 2009).
Dipyridamole	PDE 8	
PF-04447943	PDE9 inhibitor	PF-04447943 is a powerful, selective brain penetrant PDE9 inhibitor that improved cognitive function and raised indications of hippocampal synaptic plasticity in a range of cognition models in rats and mice (Hutson et al., 2011).
Mp-10 (PF-2545920)	PDE 10A inhibitor	The selective antagonist MP-10 inhibits phosphodiesterase 10A, which stimulates dopamine D2 receptor-expressing medium spiny neurons more than D1 receptor-expressing neurons (Wilson et al., 2015).
Zaprinast and dipyridamole	PDE 11 inhibitor	PDE11A is sensitive to non-selective PDE inhibitors, as well as zaprinast and dipyridamole, inhibitors that are thought to be more specific for cGMP-selective PDEs (Fawcett et al., 2000).
Tacrine	PDE	Substantial cAMP PDE inhibition (Curley et al., 1984).

## Phytochemicals for the inhibition of phosphodiesterases

The growing number of Alzheimer’s patients, along with the aging population, necessitates the development of novel treatment strategy and management (Sarma et al., 2016). The amyloid (Aβ) hypothesis has primarily guided the search for effective AD management, with the primary goal of lowering the number of senile plaques, however, with limited success to date (García-Osta et al., 2012). There is a growing consensus that existing AD therapy options start far too late to substantially decrease the disease progression or delay the emergence of the most severe symptoms (Yiannopoulou and Papageorgiou, 2013). Specific phosphodiesterase (PDE) inhibitors (Table 2) have been demonstrated to improve memory function in a variety of animal models of AD (Xia et al., 2009; Bliss and Cooke, 2011; Heckman et al., 2015; Yan et al., 2016).

Several molecules penetrate the blood-brain barrier and reach concentrations high enough to inhibit PDEs in neurons and glial cells, suggesting that their peripheral activity may have an impact on the CNS (Sallustio and Studer, 2016). Tadalafil may be present in low micromolar quantities in the brain at levels often used in animal models of AD, sufficient to inhibit both PDE5 and PDE11 (Heckman et al., 2015). Increased levels of cAMP and/or cGMP in CNS cells are most likely the major mechanism of action of PDE inhibitors. This result leads to persistent and/or increased activation of signaling pathways that affect neuroprotection/neurodegeneration processes, in addition to replenishing the low levels of cyclic nucleotides present in the elderly brain (García-Osta et al., 2012; Berridge, 2014).

### Phytochemicals that inhibit phosphodiesterase

Natural compounds, or small molecules, that operate as dual phosphodiesterase inhibitors would be useful to develop novel anti-neurodegenerative and neuroprotective drugs in the context of the multi-target directed ligand method (Ribaudo et al., 2020, 2021; Table 3). Compounds such as xanthines, alkaloids, flavonoids, coumarins, and polyphenolic acids (Figure 1), are attractive scaffolds for future optimization (Kumar et al., 2015; Mohamed et al., 2019).

Cyclic nucleotide has been shown to play an important role in cognition and motility. Changes in the levels of cyclic nucleotides, such as cAMP and cGMP, have been shown to occur in a variety of neurological disorders, including Alzheimer’s disease (Francis et al., 2011). Phosphodiesterase inhibitors are useful in the treatment of erectile dysfunction and have been proposed as a potential target site for the therapy of a variety of peripheral and neurological illnesses, including asthma, COPD, and CVS (Chronic Villus Sampling) (Maurice et al., 2014; Padda and Tripp, 2021). Rolipram, a selective PDE4 inhibitor, and its synthetic equivalents have been shown be effective for Alzheimer’s patients (Wang et al., 2020).

**FIGURE 4:**
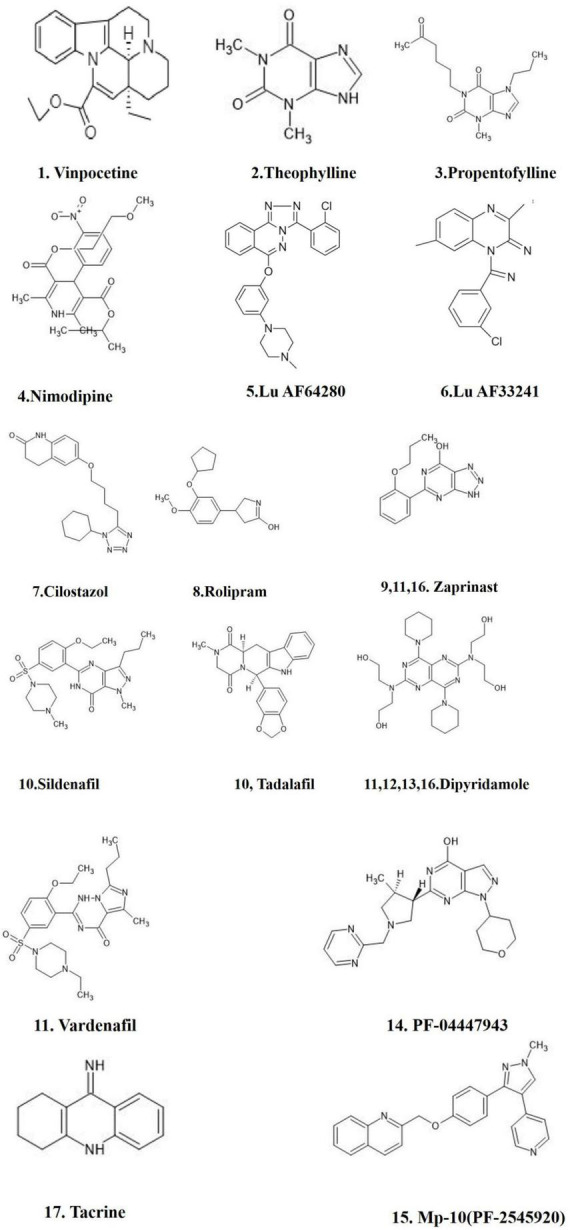
FDA approved PDE inhibitors for the treatment of AD. The figure is original and generated by Bio-render software.

Various synthetic PDE inhibitors have been used in clinical trials for a variety of illnesses and have been recommended as potential treatment options for neurological disorders (Yang et al., 2019; Table 4). However, the synthetic PDE inhibitors adverse effects have limited their usage in clinical practice. Natural products ingredients are widely accepted and regarded harmless (Pratap and Shantaram, 2020; Pratap et al., 2021). Several plant derived components have been investigated pre-clinically for PDE inhibitory action throughout the last decade (Temkitthawon et al., 2011), and they were proved to be as effective as synthetic drugs in inhibiting PDEs (Figure 4). Inhibition of specific PDEs and accumulation of cGMP may inhibit neuroinflammation and improve synaptic plasticity and memory. The PI3K/Akt pathway is enhanced by an increased cGMP levels through the suppression of PDE activity. By activating PI3K/Akt, the NO/cGMP/PKG/CREB/BDNF pathway plays a crucial part in neurogenesis and synaptic plasticity (Peixoto et al., 2015). We discuss potential plant derived compounds with PDE inhibitory activity, as well as their possible relevance in Alzheimer’s disease.

### Role of phytochemicals in the pathology of Alzheimer’s disease

Although some therapeutic medications failed at various phases of clinical testing, the increase of remedial approaches for mitigating AD progression has been encouraging (Pratap and Shantaram, 2020). The majority of these therapeutic medications inhibit only one of the numerous pathways involved in AD, which explains their lack of efficacy (Wen et al., 2015). AD is caused by the impairment of several diverse and important biological pathways. Potential AD-treating drugs must acts on multiple disease pathways to improve the efficacy (Guo et al., 2020). Natural products, small compounds, and peptidomimetics have been indicated to inhibit PDEs, prevent Aβ, and tau aggregation (Nalini et al., 2021) and expected to improve the clinical efficacy by enhancing PDE inhibitory activity in the brain, increasing the β- and α-secretase, and kinase activity (Guzior et al., 2015).

Targeting several aspects of the network causing AD could offer better benefit as compared with monotherapies (Zimmermann et al., 2007). Recent studies suggests a novel therapeutic method to treat AD, which targets two independent but synergistic pathways connected to different parts of the disease for the first time (Salloway et al., 2020). Combining HDAC (Histone Deacetylase) inhibitor with the inhibition of phosphodiesterase-5 (PDE5), an enzyme that targets another intracellular pathway has been proposed to be implicated in memory formation and other AD-related symptoms (Cuadrado-Tejedor et al., 2015). The combination of cholinesterase inhibitors with Memantine is well tolerated and safe, and is effective for individuals with moderate to severe Alzheimer’s disease (Epperly et al., 2017). Combinations of diverse disease-modifying medications with different mechanisms, on the other hand, may have potential synergic effects and boost cognition, behavior, and daily living function, except adjuvant therapies of conventional drugs (Fan and Chiu, 2014).

A 3-week chronic therapy with sub-effective levels of the cAMP-specific PDE4-I Roflumilast (0.01 mg/kg) and the cGMP-specific PDE5-I Vardenafil (0.1 mg/kg) improved recognition, spatial, and contextual fear recall, as another combination therapy (Gulisano et al., 2018). Hu et al. used the benzyl piperidine moiety of Donepezil in combination with the pyrazolopyrimidinone structure of a previously known PDE9A inhibitor to study the effects of dual PDE9A/AChE inhibitors (Maramai et al., 2022). Although several amidic or (cyclic)amine chains were investigated as linkers for the two pharmacophores, the greatest results were obtained with 4-member ethereal or carbon tethers, which resulted in compounds with submicromolar inhibitory action against PDE9A and AChE (Huang et al., 2015). Hybridization of the Pyrazolopyrimidinone skeleton with Rivastigmine yielded another series of potential PDE9A inhibitors using a similar strategy (Maramai et al., 2022).

Cuadrado-tejedor et al. (2018) investigated the effect of a simultaneous inhibitor of HDAC6 and PDE5, specifically compound CM-414, on HDACs and other AD-related proteins. The combination of two medications that target these two enzymes (Vorinostat and Tadalafil) has shown *in vivo* favorable benefits, relieving cognitive deficits in AD animals and reducing hippocampus neuron density (Cuadrado-tejedor et al., 2018; Maramai et al., 2022).

Hu et al. (2019) provided a good example of PDE inhibitors with multifunctional effectiveness against Aβ-induced toxicity and metal-chelating/antioxidant characteristics. They developed hybrid molecules that combine chloroquine’s metal ion chelating framework with critical binding site fragments from the known PDE4 inhibitors Rolipram and Roflumilast, both of which have been studied in preclinical AD models (Maramai et al., 2022).

## Summary and future prospects

Extensive experimental and clinical studies suggest that pharmacological targets involved in inhibition of PDEs could be potential therapeutic targets for cognitive impairments and dementia. PDE inhibitors modulate the cyclic purine nucleotide levels and potentially prevent or cure AD, MCI, and dementia. Natural products have been shown to improve dementia, cognitive decline, and AD, symptoms suggesting that PDE inhibitors could help prevent the disease. Dysregulation of several cellular processes, including the immune system, transduction and transcription signaling pathways, and the inflammatory response, may result from abnormality in PDEs’ physiological activity, which play important roles in neurodegenerative disorder. Various PDE inhibitors, including synthetic, natural, and multifactorial modifying and combination therapy have been discussed in this review article. We described that several neuro-psycho-pharmacological pathways are implicated in the therapeutic impact of PDEI in AD. Protective effects of such medications, which are directly related to PDE inhibition and other pharmacological effects of the pharmaceuticals, such as neurotransmitter receptor activity or certain downstream effects, may also be striking (vasodilation) have been discussed. PDEs are emerging as viable targets for the development of new pharmacotherapeutic drugs to treat AD. A well-designed clinical trials are needed to assess the efficacy and safety of PDE inhibitors in Alzheimer’s patients. Researchers can gain insights on the growth of multifunctional drugs for AD patients. With such types of drugs, AD patients would have balanced Aβ and tau balance in the brain, normal PDE and kinase activity levels. A wide range of natural compounds has been used for different diseases like cancer, AD, neuroprotective study, anti-diabetic etc. but very few plant-based compounds are used for PDEI study. Some flavonoids and Phenolic compounds have also been proven to inhibit PDEs, as evidenced by red grape extract’s *in vitro* suppression of cGMP-specific PDEs. Synthetic drugs show several side effects including flush, nausea, dizziness, dry mouth, transitory hypo- and hypertension, headache, and heartburn as compared to natural compounds. These natural compounds could be a potential candidate for developing as an alternative strategy for PDEs inhibition in AD.

## Author contributions

HF conceived the idea of the review. JS, SZ, LW, YL, and PG collected the literature, analyzed, outlined, and drafted the manuscript. HF and GK revised and finalized the manuscript. All authors participated in the study and read and approved the final manuscript.

## Abbreviations

AD: Alzheimer’s Diseases
cAMP: Cyclic Adenosine Monophosphate
cGMP: cyclic guanosine monophosphate
COPD: Chronic Obstructive Pulmonary Disease
CREB: cyclic AMP response element-binding protein
CVS: Chronic Villus Sampling
HDAC: Histone Deacetylase
MCI: Mild Cognitive Impairment
PDE-I: phosphodiesterase inhibitors
PDEs: Phosphodiesterases
PKA: Protein kinase A
PKG: Protein kinase G.
